# *Isodon rugosus* (Wall. ex Benth.) Codd In Vitro Cultures: Establishment, Phytochemical Characterization and In Vitro Antioxidant and Anti-Aging Activities

**DOI:** 10.3390/ijms20020452

**Published:** 2019-01-21

**Authors:** Bilal Haider Abbasi, Aisha Siddiquah, Duangjai Tungmunnithum, Shankhamala Bose, Muhammad Younas, Laurine Garros, Samantha Drouet, Nathalie Giglioli-Guivarc’h, Christophe Hano

**Affiliations:** 1 Department of Biotechnology, Quaid-i-Azam University, Islamabad 45320, Pakistan; aisha_siddiquah@yahoo.com (A.S.); pk.younas@gmail.com (M.Y.); 2 Laboratoire de Biologie des Ligneux et des Grandes Cultures (LBLGC EA1207), INRA USC1328, Plant Lignans Team, Université d’Orléans, 45067 Orléans CÉDEX 2, France; duangjai.tun@mahidol.ac.th (D.T.); laurine.garros@etu.univ-orleans.fr (L.G.); samantha.drouet@univ-orleans.fr (S.D.); 3 Bioactifs et Cosmétiques, GDR 3711 COSM’ACTIFS, CNRS, 45067 Orléans CÉDEX 2, France; 4 EA2106 Biomolécules et Biotechnologies Végétales, Université de Tours, 37200 Tours, France; shankhamala85@gmail.com (S.B.); nathalie.guivarch@univ-tours.fr (N.G.-G.); 5 Department of Pharmaceutical Botany, Faculty of Pharmacy, Mahidol University, 447 Sri-Ayuthaya Road, Rajathevi, Bangkok 10400, Thailand; 6 Institut de Chimie Organique et Analytique, ICOA UMR7311, Université d’Orléans-CNRS, 45067 Orléans CÉDEX 2, France

**Keywords:** *Isodon rugosus*, pentacyclic triterpenoid, phenolic acid, plant growth regulators, anti-aging, antioxidant

## Abstract

*Isodon rugosus* (Wall. ex Benth.) Codd accumulates large amounts of phenolics and pentacyclic triterpenes. The present study deals with the in vitro callus induction from stem and leaf explants of *I. rugosus* under various plant growth regulators (PGRs) for the production of antioxidant and anti-ageing compounds. Among all the tested PGRs, thidiazuron (TDZ) used alone or in conjunction with α-napthalene acetic acid (NAA) induced highest callogenesis in stem-derived explants, as compared to leaf-derived explants. Stem-derived callus culture displayed maximum total phenolic content and antioxidant activity under optimum hormonal combination (3.0 mg/L TDZ + 1.0 mg/L NAA). HPLC analysis revealed the presence of plectranthoic acid (373.92 µg/g DW), oleanolic acid (287.58 µg/g DW), betulinic acid (90.51 µg/g DW), caffeic acid (91.71 µg/g DW), and rosmarinic acid (1732.61 µg/g DW). Complete antioxidant and anti-aging potential of extracts with very contrasting phytochemical profiles were investigated. Correlation analyses revealed rosmarinic acid as the main contributor for antioxidant activity and anti-aging hyaluronidase, advance glycation end-products inhibitions and SIRT1 activation, whereas, pentacyclic triterpenoids were correlated with elastase, collagenase, and tyrosinase inhibitions. Altogether, these results clearly evidenced the great valorization potential of *I. rugosus* calli for the production of antioxidant and anti-aging bioactive extracts for cosmetic applications.

## 1. Introduction

According to a survey, around 70,000 important plant species are consumed for health and wellness purposes. Besides this, industry is continuously producing a large amount of bioactive compounds, but herbal medicines and phytotherapy are still in practice in many areas of the globe [1]. Species members of the genus *Isodon* (Schrader ex Bentham) Spach from *Lamiaceae* family are known universally for their economical and medicinal worth [2]. Plants from family *Lamiaceae* have been explored well for their health and wellness properties such as the treatment of hypertension, fever, rheumatism, dementia, toothache, cancer, antimicrobial, hypoglycemic, phytotoxic, antidiarrheal, anticholinesterase, lipoxygenase inhibitory, bronchodilator, and anthelmintic [3,4,5,6,7,8]. Among them, *I. rugosus* (Wall. ex Benth.) Codd is one of the most challenging and attractive choices to characterize its potential compounds and screen the biological activities that are applicable for cosmetic aspects. This medicinal plant is present in Pakistan, and widely distributed in the Northern areas of the country, especially in Gilgit; this *I. rugosus* is also recognized by various vernacular names such as sperkai, boi, and phaypush [5,6]. This medicinal plant is an aromatic shrub, its stems erect with the quadrangular branches, its leaves are opposite, broadly ovate shape with green color; leaf blade consist of small stellate dendroid hairs. Its inflorescence is Cymose, each flower is white or spotted pink or violet, bilabiate form, Nutlets fruit is an oblong shape with dark brown color. From a pharmacological point of view, this *Isodon* species is rich in bioactive compounds, with potential applications in cosmetics, as well as, traditional and modern medicine industries. *I. rugosus* is an aromatic medicinal plant containing essential oils. Previous works examined its essential oil composition by GC and GC-MS analysis [5,9,10,11]. Most of the analyzed extracts came from wild fresh living plants or dry plant materials harvested from the forest or the field [5,9,10,12]. The presence of pentacyclic triterpenes and caffeic acid phenolic derivatives have been also reported in this plant [13]. Important phytochemicals including pentacyclic triterpenoids (plectranthoic acid (PA), oleanolic acid (OA), betunilic acid (BA)), and other phenolic compounds were detected in *I. rugosus* [13]. As in many *Lamiaceae* species, both rosmarinic acid (RA) and caffeic acid (CA) are the predominant phenolic compounds that could be the reason behind the antioxidant properties of this plant [14,15,16].

Human beings have extensively exploited medicinal plants as bioactive ingredients for therapeutic and cosmetic applications since ancient times [17]. The anti-aging activities of plants have been credited to their intrinsic ability to reduce free radical damages to the skin, along with their ability to modulate the activity of many enzymes involved in aging process. For example, their capacity to inhibit elastase, hyaluronidase, or collagenase involve the cleavage of extracellular matrix components, while tyrosinase inhibition involves hyperpigmentation related to skin aging, or more recently to their capacity to activate SIRT1, which is a key regulator involved in the control of both oxidative stress response and regulation of aging processes. Pentacyclic triterpenoids have been regarded as effective enzymes inhibitors that are involved in the cleavage of the extracellular matrix components [18,19], whereas phenolic acids are described as strong antioxidants and possible potent SIRT1 activators [19,20,21]. As a potential rich source of these compounds, *I. rugosus* could be an attractive plant for cosmetic applications, however, currently this possibility has never been explored and the rationale of the possible biological activities of this plant are therefore unknown. Moreover, slow growth, slow germination, and a conventional way of harvesting large amount of wild plants have threatened this species. Therefore, alternative strategies are now required for both its conservation and usage. In this regard, in vitro culturing techniques ensure preservation of the uncommon and scarce medicinal plant species [22]. Production of secondary metabolites *via* tissue culture techniques provides imperative benefits to amplify the assembly of appropriate substances. Consequently, biotechnological strategies magnify the estimation of these bioactive phytochemicals [23,24]. Herbal products have gained attention worldwide because of their production of specialized metabolites such as phenolics and pentacyclic triterpenoids [25,26].

Some in vitro cultivation has been reported on some *Isodon* species including *I. serra* [27], *I. wiggthii* [28] or *I. amethystoides* [29]. However, until now, small number of studies are available on the establishment of in vitro cultivation, phytochemical analysis, and biological activities of the resulting cultures of *I. rugosus* with the exception of biogenic synthesis of ZnONPs from in vitro callus culture of this plant [30]. In the present study, we report the in vitro callus establishment through optimization of hormonal combination, significant accumulation of pentacyclic triterpenoids (BA, OA and PA) and phenolic compounds (CA and RA), as well as antioxidant and anti-ageing properties of the resulting extracts for future potential cosmetic applications. As per our knowledge, the current optimization report is the first to address *I. rugosus* in vitro cultures as a feasible large-scale production system of bioactive phenolics and pentacyclic triterpenoids.

## 2. Results and Discussion

### 2.1. Optimization of Callogenesis from Different Initial Explants

For the determination of callus induction frequency, stem and leaf explants from *I. rugosus* were cultured on MS medium encompassing different concentrations (1.0–5.0 mg/L) of several PGRs (TDZ, NAA and BAP) used either alone or in conjunction with TDZ, as shown in Table 1.

**Figure 1:**
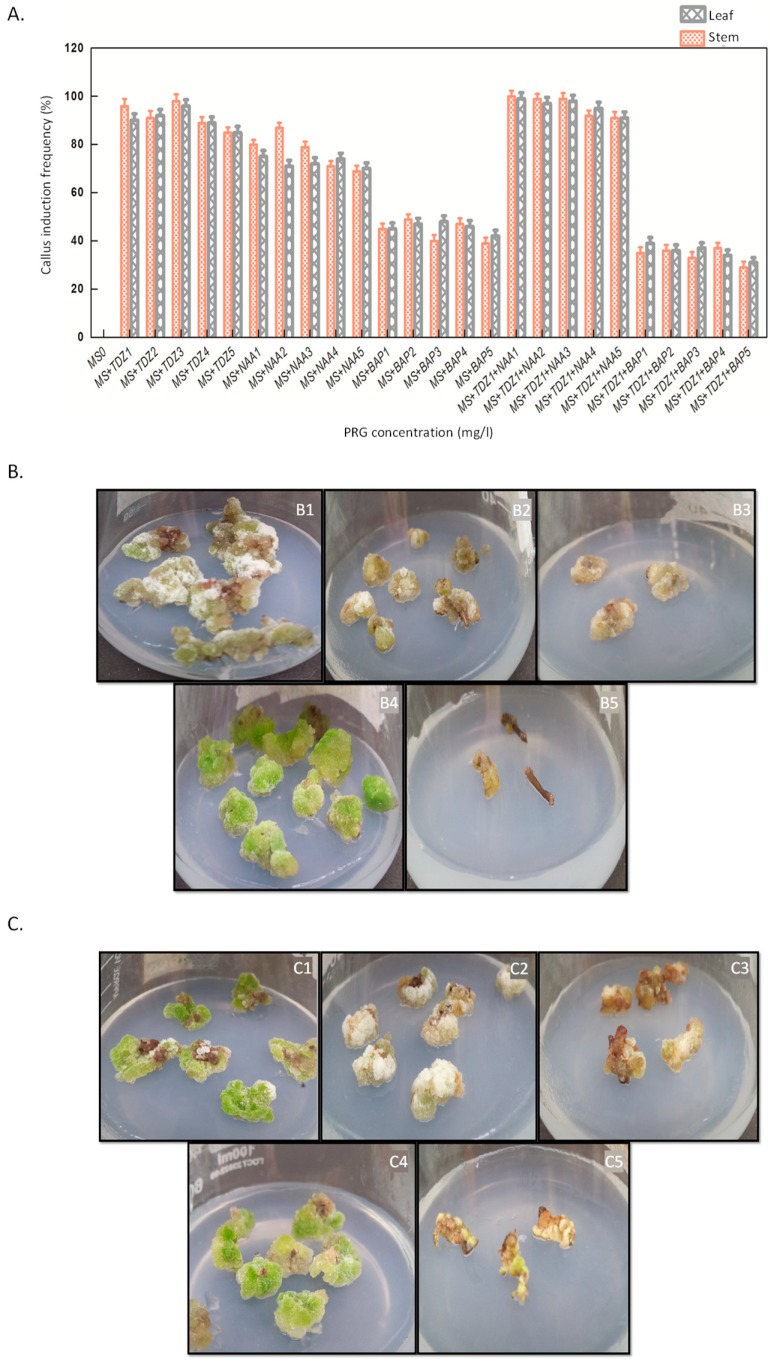
(**A**) Callus induction frequency (%) for stem and leaf explants under different PGRs concentrations. Values are means ± SE from three replicates; (**B**) Effect of 5 weeks old culture media containing various PGRs on callus induction and growth of stem explants; (**B1**) TDZ 1.0 mg/L; (**B2**) NAA 4.0 mg/L; (**B3**) BAP 2.0 mg/L; (**B4**) 1:1 TDZ 1.0 + NAA 1.0 mg/L; (**B5**) TDZ 1.0 + BAP 4.0 mg/L; (**C**) Effect of 5 weeks old culture media containing various PGRs on callus induction and growth of leaf explants; (**C1**) TDZ 3.0 mg/L; (**C2**) NAA 3.0 mg/L; (**C3**) BAP 4.0 mg/L; (**C4**) TDZ 1.0 + NAA 1.0 mg/L; and (**C5**) TDZ 1.0 + BAP 5.0 mg/L.

Callus was initiated after 10–12 days of culturing explants. In case of leaf explants, TDZ (1.0 mg/L, 2.0 mg/L and 3.0 mg/L) and 1.0 mg/L TDZ + NAA (1.0 mg/L, 2.0 mg/L and 3.0 mg/L) led to highest callus induction (95–100%) as compared to BAP alone or combination of BAP with TDZ. NAA alone resulted in around 80% callus induction, but the value greatly increased (up to 90%) when TDZ was used in combination. Similarly, the induction frequency for stem explants was close to 100% when TDZ was employed either alone or combined with NAA (Figure 1A). However, higher concentration of all the tested PGRs restricted callus induction in both stem and leaf explants, possibly due to repression of some endogenic PGRs retarding callus formation. Indeed, changes in callus response formation have already been ascribed to diverse endogenous hormonal responses pointing to the variable sensitivity of tissues toward these PGRs. Sreedevi et al. (2013) and Anjum et al. (2017) reported similar observations [31,32]. No callogenesis was observed on MS medium lacking these PGRs, which has already been observed for various other plant species such as *Stevia rebaudiana* [33].

Visual morphological variations were also detected in calli (Figure 1B,C). Generally, stem-derived calli were more friable, while leaf-derived calli were compact in texture. Similar results have previously been reported for several other medicinal plant species [34,35]. We also observed that in *I. rugosus*, the callogenic response and morphological changes were markedly influenced by the exogenously applied PGRs. Physiological response of calli also radically varied in accordance to the type of initial explant. The potential growth rate was higher in stem-derived calli, as compared to the calli derived from leaf as starting explants.

**Figure 2:**
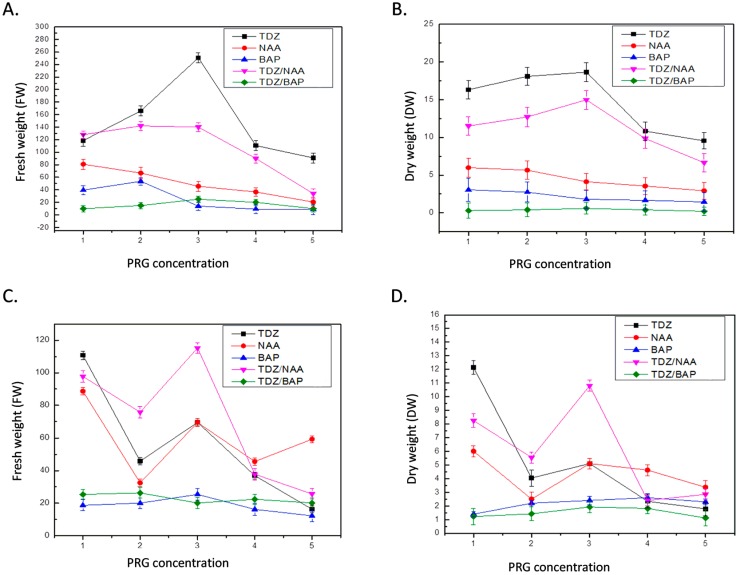
Time-course fresh and dry weight of callus cultures at different PGRs (in mg/L). (**A**) Fresh weight for stem-derived callus culture (in g/L); (**B**) dry weight for stem-derived callus culture (in g/L); (**C**) fresh weight for leaf-derived callus culture (in g/L); and (**D**) dry weight for leaf-derived callus (in g/L) cultured on MS medium fortified with TDZ, NAA, BAP (1.0–5.0 mg/L), TDZ (1.0 mg/L) + NAA (1.0–5.0 mg/L), TDZ (1.0 mg/L) + BAP (1.0–5.0 mg/L). Values are means of three replicates with standard deviation.

Murthy et al. (1998) estimated that TDZ is a potent PGR for in vitro culture successive growth [36]. TDZ at a concentration of 3.0 mg/L produced highest biomass (FW 250.65 g/L and DW 18.65 g/L) in stem-induced callus cultures (Figure 2A,B). Similarly, 1.0 mg/L TDZ + 3.0 mg/L NAA resulted in FW of 140.22 g/L and DW of 14.98 g/L. Conversely, application of BAP alone or in combination with TDZ showed least response for biomass accumulation (Figure 2A,B). As for NAA, maximum biomass (FW 80.79 g/L and DW 5.98 g/L) was observed at 1.0 mg/L concentration, which then gradually decreased with increase in the concentration of NAA, as shown in Figure 2A,B. In case of leaf-derived callus cultures, optimum biomass accumulation (FW 115.2 g/L and DW 10.81 g/L) was observed for 1.0 mg/L TDZ + 3.0 mg/L NAA (Figure 2C,D). However, BAP + TDZ resulted in minimum biomass accumulation (Figure 2C,D).

With respect to anatomical structure of callus, previous reports proved that callus induction frequency and proliferation increased considerably with the precise ratio of two exogenous hormones. Hypothetically, this was due to the effect and synthesis of endogenously grown regulators upon their exogenous presentation. Sahraoo et al. (2014) and Lee (1971) reported similar findings [37,38]. TDZ-treated tissues in combination with auxin maintain and enhance their accumulation and transport. Our data is supported by Guo et al. (2011), who confirmed that the use of TDZ alone or in collaboration with other PGRs provoke high rate of callogenesis and cell proliferation due to high intrinsic activity and low absorbance in callus [39]. The current investigation explores that despite explants used for callogenesis, maximum growth is observed with lower concentration of all PGRs, used either alone or in combination, but biomass is gradually inhibited at higher concentrations. Our data also suggests that the combined treatment of TDZ and NAA is the best for callogenesis and biomass accumulation in *I. rugosus* callus cultures.

### 2.2. Evaluation of Secondary Metabolites Production

**Figure 3:**
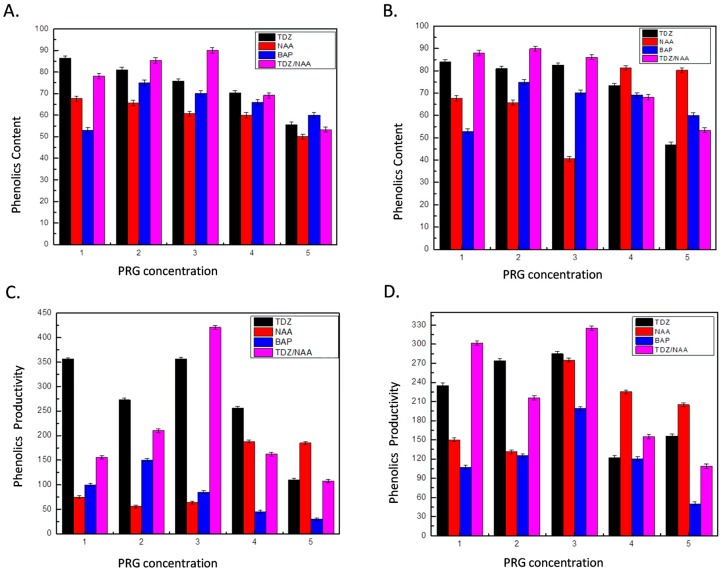
Comparison of total phenolic content (TPC) and total phenolic productivity (TPP) of extracts at different PGRs. (**A**) TPC (in mg/g DW) for stem-derived callus culture; (**B**) TPC (in mg/g DW) for leaf-derived callus culture; (**C**) TPP (in mg/L) for stem-derived callus culture; and (**D**) TPP (in mg/L) for leaf-derived callus cultured on MS medium fortified with PRG (TDZ, NAA, BAP (1.0–5.0 mg/L), TDZ (1.0 mg/L) + NAA (1.0–5.0 mg/L), TDZ (1.0 mg/L) + BAP (1.0–5.0 mg/L)). Values are means of three replicates with standard deviation.

Total polyphenols accumulation in stem-derived calli of *I. rugosus* on all the tested PGRs ranged from 49.99 to 90.06 mg/g DW (Figure 3).

Calli cultured on media supplemented with TDZ (1.0 mg/L) and NAA (3.0 mg/L) biosynthesized optimum levels (90.06 mg/g DW) of phenolic compounds (Figure 3A), while lowest accumulation (49.9 mg/g DW) was observed in media supplemented with high concentration (5.0 mg/L) of NAA. Phenolics accumulation in response to NAA and TDZ gradually declined with increases in hormonal concentration. However, Szopa, and Ekiert (2014) observed that PGRs directly influence the production of phenolic compounds in plants in vitro cultures [40]. Among all the PGRs, combined treatment of TDZ + NAA at low concentration exhibited maximum accumulation of TPC in stem-derived calli. Similar trend was observed for TPC in leaf-derived callus culture (Figure 3B) for which TDZ combined with NAA gave highest accumulation as compared to TDZ or NAA used alone. Faizal et al. (2017) reported that the best treatment for phenolic compounds production in red pitaya callus was 2.0 mg/L NAA + 4 mg/L TDZ, which is consistent with the results of our study [41]. Similarly, Tariq et al. (2014) also highlighted that growth regulators such as NAA and TDZ greatly influence the production of phenolic compounds, flavonoids, and antioxidants in *A. absinthium* cultures grown in vitro [42].

Antioxidant capacity generally correlated with TPC, thus collinear connection exists between these two variables, as evident from the literature [43,44,45]. Likewise, Khandaker et al. (2012) also indicated that the improved antioxidant activities in apple treated with different PGRs were linked with the increase in TPC [46]. A similar trend was also observed here with the quenching free radical activity (Figure S1). Our data suggests that *I. rugosus* extract could serve as a safe antioxidant agent.

The presence of pentacyclic triterpenes and caffeic acid phenolic derivatives have already been reported in this plant family [13,14,15,16]. Therefore, in order to make a step forward, the presence of these compounds were investigated in 12 callus cultures (hereafter called Ir#1 to Ir#12) grown on various culture media (precise PGRs composition of these media is shown in Supplementary Table S1) selected on the basis of DW accumulation, TPC and radical scavenging activity. A magnification showing and the quantification results are depicted in Table 2.

**Figure 4:**
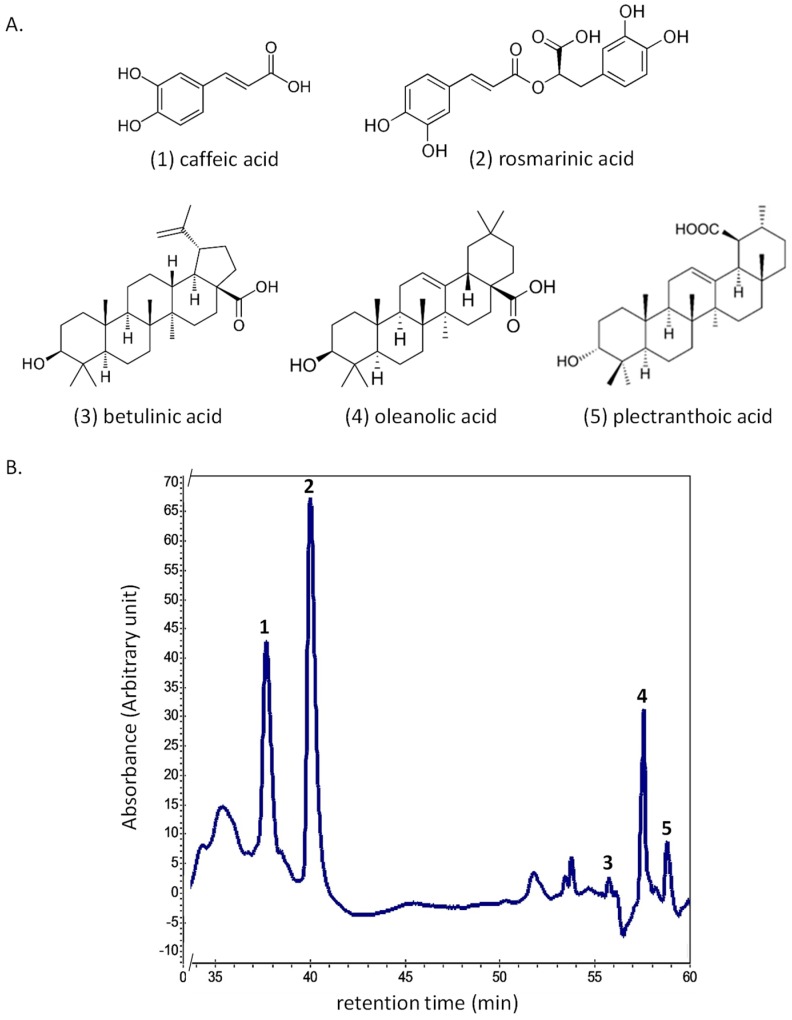
(**A**) Chemical structures of main phytochemicals accumulated in callus cultures of *I. rugosus* caffeic acid (CA, **1**) rosmarinic acid (RA, **2**) betulinic acid (BA, **3**) oleanolic acid and (OA, **4**) plectranthoic acid (PA, **5**); (**B**) Magnification of typical HPLC chromatograms showing the correct separation of the main phytochemicals accumulated in callus cultures of *I. rugosus*.

Following HPLC analysis, phenolic acids contents ranged from 488.4 (Ir#2) to 979.5 (Ir#6) µg/g DW for CA and 751.6 (Ir#2) to 2013.5 (Ir#11) µg/g DW for RA, whereas, pentacyclic triterpenoids contents ranged from 54.2 (Ir#7) to 171.2 (Ir#11) µg/g DW for BA, 198.2 (Ir#6) to 631.0 (Ir#5) µg/g DW for OA and 69.9 (Ir#9) to 454.8 µg/g (Ir#1) for PA (Figure 4). Stem-derived calli were found to be the most suitable in accumulating highest levels of different phytochemicals, except CA, while lowest amount was observed for leaf-derived calli, except BA. These results clearly evidenced that the initial explant could be an important parameter to take into account for an optimal accumulation of secondary metabolites in callus cultures of *I. rugosus*. Similarly, TDZ supplementation resulted in a higher secondary metabolites production in stem-derived callus as compared to leaf-derived callus grown on the same medium. On the contrary, TDZ combined with NAA favored the secondary metabolites production in callus initiated from leaf explants (Table 2). TDZ at lower concentration (1.0 mg/L) resulted in optimal production of PA (Ir#1), while higher concentration (3.0 mg/L) of TDZ favored maximum accumulation of CA (Ir#6) and OA (Ir#5), but interestingly the nature of the initial explant differed between these two conditions: leaf-derived callus for optimal CA production vs. stem-derived callus for optimal OA accumulation. CA and RA are regarded as major phenolic metabolites of plants from *Lamiaceae* family [47,48]. In this study, we endeavor an effort to report in vitro culture condition for accumulation and production of these phenolics and pentacyclic triterpenes metabolites in *I. rugosus* callus culture. Until now, no report is available on phytochemical composition of *I. rugosus* in vitro cultures, except GC analysis of the wild extract and characterization and composition of essential oil [11,49,50,51]. However, in vitro production and accumulation of pentacyclic triterpenes have been documented in the literature [47,52,53,54]. Fedoreyev et al. (2005) and Hagina et al. (2008) also established callus culture of *Eritrichium sericeum* that produced a higher amount of both CA and RA [55,56]. Some other studies dealing with biotechnological approaches to produce RA using plant in vitro cultures reported a higher amount of this phenolic [57]. However, our *I. rugosus* callus culture system has the advantage of producing both types of secondary metabolites. We also anticipate that elicitation strategies could further stimulate the production of these compounds in future studies.

### 2.3. Evaluation of Antioxidant and Anti-Aging Potential of I. rugosus Callus Extracts

A complete screen of antioxidant and anti-ageing capacities of these 12 extracts with contrasting phytochemical profiles was also evaluated in the current study (Figure 4A). For in vitro antioxidant screening, antioxidant mechanisms were based on both electron transfer (FRAP and CUPRAC assays) and hydrogen atom transfer (ABTS and ORAC assays) [58]. Besides these two antioxidant mechanisms involved in the scavenging of reactive oxygen species, transient metal ion chelation was also considered as an antioxidant mechanism, since the Fenton reaction, responsible for the hydroxyl radical formation, and subsequently, radical chain reaction propagation, could be inhibited through this chelating mechanism. The chelation potential of these extracts was evaluated by both FRAP and metal chelating assays using ferrozine. The results of this antioxidant screening are reported in Table 3. All of the callus extracts of *I. rugosus* exhibited marked antioxidant and chelation activities. Extract from sample Ir#11 (stem-derived calli grown on 1.0 mg/L TDZ + 3.0 mg/L NAA) displayed highest antioxidant activities for all of the assays with values of 1203.7 TEAC for DPPH, 945.8 for ABTS, 733.3 TEAC for ORAC, 535.8 for FRAP, 460.2 for CUPRAC and 54.8 µmol of fixed Fe^3+^. On the other hand, extract of sample Ir#2 (leaf-derived calli grown on 1.0 mg/L TDZ) presented the lowest antioxidant activities with values of 474.4 TEAC for DPPH, 434.5 TEAC for ABTS, 306.7 TEAC for ORAC, 211.9 TEAC for FRAP, 193.3 TEAC for CUPRAC, and 23.0 µmol of fixed Fe^3+^. Whatever the test used, ET-based assays gave higher antioxidant capacities than HAT-based assays. The prominence of this action mode suggested the occurrence of at least one phytochemical involved in this type of antioxidant mechanism in *I. rugosus* callus extracts. Here, stem-derived callus extracts displayed higher antioxidant activities than the callus initiated from leaf explants. Combination of NAA and TDZ appeared to potentially further this biological activity.

The next step involved the evaluation of anti-aging action of *I. rugosus* callus extracts (at a fixed concentration of 50 µg/mL) determined as their in vitro capacities: (1) To inhibit elastase, hyaluronidase, collagenase (Matrix Metalloproteinase type 1 (MMP1)), tyrosinase and AGEs, and (2) to activate SIRT-1 activity. Elastase, hyaluronidase and collagenase have been found to degrade extracellular matrix components in the dermis, thus leading to skin alterations including skin tonus, deep wrinkles and resilience losses [19,59,60]. Tyrosinase dysfunctions advance with aging and can lead to malignant melanoma, as well as pigmentary disorders such as freckles or melisma [61]. Oxidative stress has been found to be associated with aging and age-related diseases [62] that could lead to the buildup of advanced glycation end products (AGEs) [63]. Therefore, compounds with the ability to inhibit these enzymatic activities or processes have attracted increasing attention in cosmetics. Several studies have challenged the classical radical theory of aging [64], and SIRT-1 (a class III deacetylase) have emerged as a new key factor of longevity controlling oxidative stress effects through the stimulation of antioxidant response via FOXOs and p53 pathways [65]. A stimulation of SIRT-1 activity has been reported to be crucial in the control of oxidative stress and in the regulation of aging process [65,66]. Interestingly, phytochemicals have been reported to activate SIRT-1 homologs and to prolong life span in yeast, drosophila, and *Caenorhabditis elegans* models [20,67,68,69]. The identification of SIRT-1 activators is also of great interest for cosmetic applications.

The results are presented in Table 4, expressed as a percentage of relative activities compared to control (consisting in extraction solvent addition to the assay). Here, we have considered an inhibition percentage of 30% as a marked inhibitory effect, we have detected strong inhibitory actions of our extracts toward tyrosinase (up to 72.2% inhibition observed with the stem-derived callus extract, Ir#3), collagenase (up to 36.3% inhibition with the stem-derived callus extract, Ir#5), and a strong inhibition of AGEs formation (up to 34.1% inhibition obtained with the leaf-derived callus extract, Ir#12). Inhibitory effects observed for elastase and collagenase were less marked (up to 25.3% and 22.2% respective inhibition observed with the same leaf-derived callus extract Ir#12). Interestingly, we observed that TDZ alone was more efficient in inducing the anti-aging action in stem-derived callus through the inhibition of these enzymes; whereas, addition of NAA appeared to limit the effect of initial explant origin and even seemed to reverse it (compare Ir#11 and Ir#12, Table 4). With the exception of tyrosinase inhibition, Ir#12 extract (leaf-derived callus grown on TDZ (1.0 mg/L) and NAA (3.0 mg/L)) appeared to be the most promising extract for these anti-aging activities.

Conversely, stem-derived callus was more prompted to stimulate SIRT1 activity with a maximum 2-fold increase measured with Ir#11 extract (stem-derived callus grown on TDZ (1.0 mg/L) and NAA (3.0 mg/L)). An activation level that is very similar to the stimulatory effect measured with resveratrol (the reference activator of SIRT1) [20]. However, here we have to note that a simple extract was used that could be very interesting since no purification steps are needed to obtain this activation. Moreover, without minimizing the potential synergistic effects, we assumed that all these anti-aging actions could be further reinforced following the purification steps.

### 2.4. Correlations Analysis

**Figure 5:**
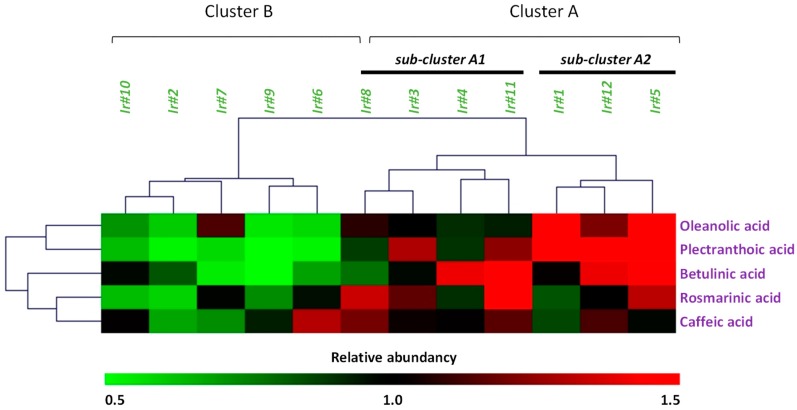
Hierarchical clustering analysis (HCA) of twelve *I. rugosus* callus extracts related to their phytochemical profile.

Hierarchical clustering analysis (HCA) was applied first to discriminate the different sample extracts based on their qualitative and quantitative phytochemical profiles (Figure 5). Regarding this HCA, decomposition into two main groups was observed. The first cluster (i.e., cluster A on Figure 5) grouped together sample extracts with the highest phytochemical accumulation capacities, with the sub-cluster A1 rich in pentacyclic triterpenes (BA, PA, and OA) and the sub-cluster A2 accumulating highest amount of phenolic acids (RA and CA). In contrast, cluster B shows sample extracts with the lowest accumulation capacities of these compounds. While we previously observed that stem-derived callus was generally more attractive than leaf-derived callus when considering each hormonal treatment individually, here the HCA pointed that the hormonal treatment is a prominent parameter over explant origin for an optimal accumulation since both stem and leaf-derived callus sample extracts were distributed equally in both clusters. Combination of NAA with higher concentrations of TDZ (Ir#11 and Ir#12) appeared to be the most favorable hormonal balances for accumulation of both pentacyclic triterpene and phenolic acid compounds in *I. rugosus* in vitro cultures.

**Figure 6:**
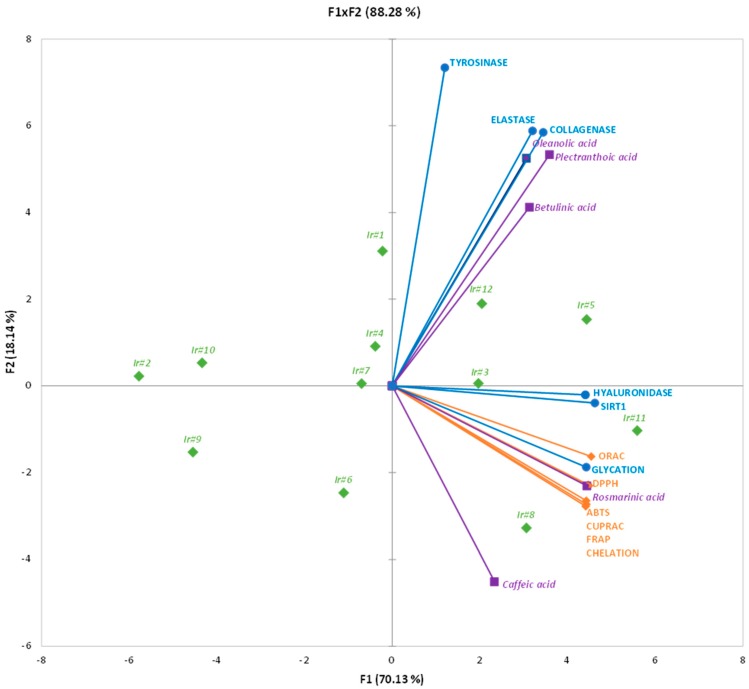
Principal component analysis (PCA) of the different phytochemicals and biological activities of *I. rugosus* callus extracts. Variance of factor 1 (F1) = 70.13% and of factor 2 (F2) = 18.14%.

In order to rationale the apparent complex linkage between phytochemicals and biological activities, a principal component analysis (PCA) was then conducted (Figure 6). The obtained separation was satisfactory and allowed explaining 88.28% of the apparent complexity (F1×F2, Figure 6). The discrimination mainly occurred through the first dimension (F1 axis), explaining 70.13% of the apparent complexity by itself and allowing the separation of the sample extracts according to their phytochemical composition and biological activities. This point is of particular interest since it clearly evidences that it is possible to predict the antioxidant and anti-aging potential of a sample extract based on its phytochemical profile and that a direct linkage could exist between these two parameters. The second dimension axis (F2) accounted for only 18.14% of the initial variability, but intriguingly, it allowed the discrimination between: 1) Sample extracts rich in pentacyclic triterpenes and showing high inhibition capacities of tyrosinase, elastase and collagenase, and 2) sample extracts rich in phenolic acids showing the highest antioxidant activities, anti-AGEs, hyaluronidase inhibition, and SIRT1 activation. From this analysis, it appeared that sample extracts Ir#8 and Ir#11 were the most attractive for cosmetic applications looking for natural antioxidants, anti-hyaluronidase, anti-AGEs, and/or activator of SIRT1, whereas sample extracts Ir#1, Ir#2, and Ir#5 were the most promising for a natural anti-tyrosinase, anti-elastase, and/or anti-collagenase applications. Note that sample extracts Ir#3 appeared as the most potent extract for these cosmetic applications targeting the whole set of these activities.

To better assess the linkage between individual phytochemical and biological activities, Pearson coefficient correlations (PCCs) between these parameters were also calculated (Table 5). From this analysis, it appeared that the phenolic acid RA is the main contributor towards the antioxidant activities of *I. rugosus* in vitro cultures, with high (ranging from 0.982 for ABTS assay to 0.997 for DPPH and FRAP assays) and highly significant (*p* < 0.001) PCCs (Table 5). The anti-AGEs activity correlated with the presence of phenolic acids RA (PCC = 0.943, *p* < 0.001), and to a lesser extent, CA (PPC = 0.608, *p* = 0.036) could be linked to their well-described antioxidant activity [55]. Furthermore, it was observed that the pentacylic triterpene PA also significantly contributed towards the antioxidant ORAC assay (PCC = 0.604, *p* = 0.038). Concerning anti-aging activities, the analysis revealed a more complex linkage. The marked activation of SIRT1 activity and the anti-hyaluronidase activity appeared to be relied on RA and pentacylic triterpenes. Phenolic compounds are known as potent activator of SIRT1 activity [20], whereas both phenolic compounds and triterpenes are described as possible hyaluronidase inhibitors [70]. The marked anti-tyrosinase activity of our sample extracts was significantly linked with PA (PCC = 0.622, *p* = 0.031) and OA (PCC = 0.603, *p* = 0.038), but not with the third pentacylic terpene BA. From a structural point of view, PA and OA originate from the same olenyl cation precursor [71], which could be one explanation of this observation. The marked anti-collagenase, as well as less pronounced anti-elastase activities of *I. rugosus* sample extracts were correlated significantly with the pentacylic triterpenes.

## 3. Materials and Methods

### 3.1. Chemicals and Reagents

All the extraction solvents employed in this study were of analytical grade, provided by Thermo Scientific (Illkirch, France). Standards and reagents were obtained from Sigma-Aldrich (Saint-Quentin Fallavier, France).

### 3.2. Plant Material

The seeds of *I. rugosus* were obtained from wild plant species located in Khyber Pakhtunkhwa province of Pakistan in December 2015. Seeds were surface sterilized prior to culturing in order to escape contamination. The air-dried seeds were then germinated on Murashige and Skoog (MS) (1962) medium comprising 0.8% agar and 30 g/L sucrose [72]. The pH of all the media was maintained at 5.8 before autoclaving at 121 °C for 20 min. For the establishment of callogenesis, stem and leaf explants were cut out from four weeks old in vitro grown plantlets of *I. rugosus* and cultured at various concentrations of PGRs, either alone or in combination.

### 3.3. Callogenic Frequency

Three different PGRs (NAA, TDZ and BAP) at varied concentrations (1.0–5.0 mg/L), used either alone or in combination with 1.0 mg/L TDZ, were employed for callus induction in the present study. The explants were maintained in a growth room for 16/8 h (light/dark cycle) at 25 ± 1 °C. Observation of callogenic frequency and callus morphology was done on weekly basis with visual eye. Respective calli were then sub-cultured on fresh medium supplemented with the same PGRs concentrations after every four weeks of the culture. Fresh weight and dry weight were also determined for subsequent examinations.

### 3.4. Determination of Total Phenolic Compounds Content

For phytochemical screening, extraction from calli was done according to the method presented by Ali et al. (2013) [73]. Briefly, 100 mg of dried callus was added to 10 mL of methanol (80%) to prepare the extract. The samples were then sonicated for 10–15 min, followed by vortexing for five–seven min and the procedure was repeated twice. After centrifugation at 6000 rpm for 15 min, the supernatants were collected and kept for further analysis.

Procedure described by Velioglu et al. (1998) was adopted for the estimation of total phenolic content (TPC) using FC reagent [74]. In brief, 20 μL of sample and l80 μL of FC reagent were mixed and incubated for five min. Then, 90 μL of sodium carbonate was added to the mixture and the OD was recorded at 630 nm with the help of a microplate reader. The calibration curve (0–50 μg/mL, R2 = 0.968) was generated by using gallic acid as a standard and the TPC was expressed as gallic acid equivalents (GAE) per gram of dry weight (DW) sample. Total phenolic production (TPP) was examined by using the following equation: TPP (mg/L) = TPC (mg/g) × Dry Weight (g/L).

### 3.5. Quantification and Identification by HPLC

The contents of caffeic acid, rosmarinic acid, oleanolic acid, plectranthoic acid and butelinic acid were determined by HPLC. Following a published protocol, a reversed-phase HPLC equipped with autosampler, Varian (Les Ulis, France) Prostar 230 pump and a Varian Prostar 335 photodiode array detector was used and controlled with Galaxie software (Varian v1.9.3.2) [19]. Briefly, the separation was achieved on an RP-18 column (5 μm, 250 × 4.0 mm id (Purospher Merck, Fontenay sous Bois, France)) at 35 °C. The mobile phase was comprised of acetonitrile (as solvent A) and 0.1% (*v*/*v*) formic acid acidified ultrapure water (as solvent B). The composition of the mobile phase varied during a 60 min run according to a linear gradient ranging from a 5:95 (*v*/*v*) to 100:0 (*v*/*v*) mixture of solvents A and B, respectively, at a flow rate of 0.6 mL/min. Detection was accomplished at 210 nm and 254 nm. Quantification and identification of each compound was done by coupling with retention times, UV spectra to those of authentic reference standards and by standard additions. Calibration curve (six points) was used to quantify each standard with the range of 0.05–1 mg/mL and correlation coefficient of at least 0.9994.

### 3.6. Antioxidant DPPH Assay

For this assay, 20 μL of each sample extract was combined with 180 μL of DPPH reagent and absorbance was recorded at 517 nm with the help of a microplate reader. The following equation was then used to calculate DPPH activity: % scavenging = 100 × (Abc-Abs/Abc).

Where, Abc denotes absorbance of the control, while Abs is absorbance of the sample or expressed as TAEC (Trolox C equivalent antioxidant capacity).

### 3.7. Antioxidant ORAC Assay

Oxygen radical absorbance capacity (ORAC) assay was carried out as suggested by Prior et al. (1998) [58]. In brief, 10 μL of the extracted sample was mixed with 190 μL of 0.96 µM fluorescein in 75 mM phosphate buffer (pH 7.4) and incubated for about 20 min at 37 °C. Then, 20 µL of 119.4 mM 2,2′-azobis-amidinopropane (ABAP) was added and the fluorescence intensity was recorded every five min during 2.5 h at 37 °C with the help of a fluorescence spectrophotometer (BioRad, Marnes-la-Coquette, France) set with an excitation at 485 nm and emission at 535 nm. Assays were made in triplicate and antioxidant capacity determined using ORAC assay was expressed as TAEC.

### 3.8. Antioxidant ABTS Assay

ABTS assay was accomplished with the method of Velioglu et al. (1998) [74]. Briefly, 2,2-azinobis (3-ethylbenzthiazoline-6-sulphonic acid (ABTS) solution was made by mixing equal proportion of ABTS salt (7 mM) with potassium persulphate (2.45 mM) and the mixture was kept in the dark for 16 h. The absorbance of the solution was measured at 734 nm and adjusted to 0.7 and then mixed with the extracts. The mixture was again kept in the dark for 15 min at 25 ± 1 °C and the absorbance was recorded at 734 nm using a BioTek ELX800 Absorbance Microplate Reader (BioTek Instruments, Colmar, France). Assays were made in triplicate and antioxidant capacity was expressed as TAEC.

### 3.9. Antioxidant FRAP Assay

Modified method of Benzie and Strain (1996) was used for the determination of ferric reducing antioxidant power (FRAP) assay [75]. In brief, 10 μL of the extracted samples were mixed with 190 μL of FRAP solution (composed of 20 mM FeCl3, 10 mM TPTZ, 6H_2_O and 300 mM acetate buffer (pH 3.6) in a ratio 1:1:10 (*v*/*v*/*v*)). Reaction mixtures were then incubated at 25 ± 1 °C for 15 min. Absorbance of the reaction mixture was noted at 630 nm using a BioTek ELX800 Absorbance Microplate Reader (BioTek Instruments). Assays were made in triplicate and the antioxidant capacity, determined using this assay, was expressed as TAEC.

### 3.10. Antioxidant CUPRAC Assay

Cupric ion reducing antioxidant capacity (CUPRAC) assay was evaluated by some modifications in the method of Apak et al. (2004) [76]. Briefly, 10 μL of samples and 190 μL of CUPRAC reaction solution (containing 7.5 mM neocuproine, 10 mM Cu(II) and 1 M acetate buffer (pH 7) in a ratio 1:1:1 (*v*/*v*/*v*)) were mixed. Reaction mixtures were then incubated at 25 ± 1 °C for 15 min and absorbance was recorded at 450 nm using a BioTek ELX800 Absorbance Microplate Reader (BioTek Instruments). Assays were made in triplicate and antioxidant capacity determined using this assay was expressed as TAEC.

### 3.11. Metal Chelating Activity Assay

The ferrous ion chelating activity of *I. rugosus* extracts was evaluated following a slightly modified method of Srivastava et al. (2012) [77]. In brief, 10 µL of extracts were mixed with ferrous iron at a final concentration of 50 μM in HEPES (pH 6.8) buffers and 50 µL ferrozine (5 mM aqueous solution). All experiments were performed in a 96-well microplates at room temperature (25 ± 1 °C). Each sample was measured with and without (blank) the addition of ferrozine. Absorbance was noted at 550 nm instantaneously after addition of ferrozine and five min later with a BioTek ELX800 Absorbance Microplate Reader (BioTek Instruments). Chelating activity values were expressed in µM of fixed Fe^3+^.

### 3.12. Collagenase Assay

Collagenase clostridium histolyticum (Sigma Aldrich) was employed for this assay and its activity was determined with the aid of a spectrophotometer by making use of *N*-[3-(2-furyl)acryloyl]-Leu-Gly-Pro-Ala (FALGPA; Sigma Aldrich) as a substrate in accordance to the protocol of Wittenauer et al. (2015) [78]. The absorbance decrease of FALGPA was followed at 335 nm for 20 min using a microplate reader (BioTek ELX800; BioTek Instruments, Colmar, France). Triplicated measurements were used and the anti-collagenase activity was revealed as a % of inhibition relative to corresponding control (adding same volume of extraction solvent) for every extract.

### 3.13. Elastase Assay

Elastase assay was performed by using porcine pancreatic elastase (Sigma Aldrich) and its activity was determined with spectrophotometer by making use of *N*-Succ-Ala-Ala-Ala-*p*-nitroanilide (AAAVPN; Sigma Aldrich) as a substrate and following p-nitroaniline release at 410 nm using a microplate reader (BioTek ELX800; BioTek Instruments) by adopting the method of Wittenauer et al. (2015) [78]. Triplicated measurements were used and the anti-elastase activity was expressed as a % of inhibition relative to the corresponding control (adding same volume of extraction solvent) for every extract.

### 3.14. Hyaluronidase Assay

Hyaluronidase inhibitory assay was carried out as suggested by Kolakul et al. (2017) using 1.5 units of hyaluronidase (Sigma Aldrich) and hyaluronic acid solution (0.03% (*w*/*v*)) as substrate [79]. The precipitation of undigested form of hyaluronic acid occurred with acid albumin solution (0.1% (*w*/*v*) BSA). The absorbance was measured at 600 nm using a microplate reader (BioTek ELX800; BioTek Instruments, Colmar, France). The hyaluronidase inhibitory effect was expressed as a % of inhibition relative to the corresponding control (adding same volume of extraction solvent) for every extract.

### 3.15. Tyrosinase Assay

Method of Chai et al. (2018) was used for the determination of tyrosinase assay [80]. In brief, L-DOPA (5 mM; Sigma Aldrich) was used as diphenolase substrate and mixed in sodium phosphate buffer (50 mM, pH 6.8) with 10 µL of *I. rugosus* extract. Finally, 0.2 mg/mL of mushroom tyrosinase solution (Sigma Aldrich) was added to this mixture to make a final volume of 200 µL. Control, with an equal amount of extraction solvent replacing the extract, was routinely carried out. The reaction processes were traced by using a microplate reader (BioTek ELX800; BioTek Instruments) at a wavelength of 475 nm. The tyrosinase inhibitory effect was expressed as a % of inhibition relative to the corresponding control for each extract.

### 3.16. Anti-AGE Formation Activity

The inhibitory capacity of AGE formation was determined as described by Kaewseejan and Siriamornpun (2015) [81]. *I. rugosus* extracts were mixed with 20 mg/mL BSA (Sigma Aldrich) solution prepared in 0.1 M phosphate buffer (pH 7.4), 0.5 M glucose (Sigma Aldrich) solution prepared in phosphate buffer and 1 mL of 0.1 M phosphate buffer at pH 7.4 containing 0.02% (*w*/*v*) sodium azide. This mixture was incubated at 37 °C for five days in the dark and then the amount of fluorescent AGE formed was determined using a fluorescence (VersaFluor fluorometer; Bio-Rad, Marnes-la-Coquette, France) set with 330 nm excitation wavelength and 410 nm emission wavelength. The percentage of anti-AGEs formation was revealed as a % of inhibition relative to the corresponding control (adding same volume of extraction solvent) for every extract.

### 3.17. SIRT-1 Assay

SIRT-1 activity was determined using the SIRT1 Assay Kit (Sigma Aldrich) following manufacturer instructions and fluorescent spectrometer (Biorad VersaFluor, Marnes-la-Coquette, France) set with 340 nm excitation and 430 nm emission wavelengths. The relative SIRT1 activity was revealed as a percentage relative to the corresponding control (adding same volume of extraction solvent) for every extract.

### 3.18. Statistical Analysis

Each experiment was carried out in triplicate and XL-stat_2018 (Addinsoft, Paris, France) was used for statistical analysis.

## 4. Conclusions

Our results hypothesized that cell culture protocols provide an excellent reproducible opportunity to optimize and obtain a uniform and high quality yield of the target compounds. HPLC analyses confirmed the presence of pentacyclic triterpenes namely plectranthoic acid (PA), betulinic acid (BA) and oleanolic acid (OA) and phenolic acids like caffeic acid (CA) and rosmarinic acid (RA) in all in vitro callus culture conditions. The impact of TDZ and NAA, as well as, the origin of initial explant phytochemical accumulation of the resulting *I. rugosus* was elucidated and correlated with relevant biological activities. Little is known about the in vitro biosynthesis, regulation, and accumulation of triterpenes and phenolic compound of *Isodon* genera. Hence, present research emphasizes a possible connection with respect to morphology, growth behavior and metabolic activity to produce fast-growing friable calli that is constantly able to generate the bulk of the target substances. Results showed the possibility to produce very contracting sample extracts in term of both phytochemical profiles and biological activities relying on simple and reproductive initial conditions. Taking advantage of these contrasting accumulation profiles, we have shown that *I. rugosus* in vitro cultures could represent a very promising and sustainable system for the production of anti-aging and antioxidant extracts for cosmetic applications. Correlation analysis further helped us to elucidate the complex link connecting phytochemicals accumulated in the callus to the biological activities of the resulting sample extracts. The antioxidant, anti-glycation, and SIRT1 activation actions relied on the presence of RA, whereas anti-tyrosinase, anti-elastase, and anti-collagenase activities were found to be linked with the occurrence of pentacylic triterpene derivatives. We anticipate that the methodology employed here could be applied to other health promoting activities of these extracts from *I. rugosus* in vitro cultures and to other plant production systems. Our research will facilitate, in the future, to enhance and examine the production of these bioactive metabolites on large-scale cultivation in bioreactors involving several biotechnological strategies like plant cell, tissue, and organ cultures.

## Figures and Tables

**Table 1:** Callogenesis, initiation (day) and morphology of stem and leaf callus under different PGRs after 5 weeks of culture.

S. No.	Treatment (mg/L)	Callus Initiation (day)		Callus Induction Frequency (%)	Callus Color	Callus Texture		Degree of Callus Formation
		Stem	Leaf			Stem	Leaf	
0	Control (MS0)	-	-	-	-	-	-	-
1	MS + TDZ 1.0	10	14	80–100	DG	F	C	+++
2	MS + TDZ 2.0	10	14	80–100	DG	F	C	+++
3	MS + TDZ 3.0	10	14	80–100	DG	F	C	+++
4	MS + TDZ 4.0	10	14	80–100	DG	F	C	+++
5	MS + TDZ 5.0	10	14	80–100	DG	F	C	+++
6	MS + NAA 1.0	12	12	40–60	SG	F	C	++
7	MS + NAA 2.0	12	12	40–60	SG	F	C	++
8	MS + NAA 3.0	12	12	40–60	SG	F	C	++
9	MS + NAA 4.0	12	12	40–60	SG	F	C	++
10	MS + NAA 5.0	12	12	40–60	SG	F	C	++
11	MS + BAP 1.0	20	20	20–30	SLG	F	C	+
12	MS + BAP 2.0	20	20	20–30	SLG	F	C	+
13	MS + BAP 3.0	20	20	20–30	SLG	F	C	+
14	MS + BAP 4.0	20	20	20–30	SLG	F	C	+
15	MS + BAP 5.0	20	20	20–30	SLG	F	C	+
16	MS + TDZ 1.0 + NAA 1.0	8	8	90–100	FG	F	C	+++
17	MS + TDZ 1.0 + NAA 2.0	8	8	90–100	FG	F	C	+++
18	MS + TDZ 1.0 + NAA 3.0	8	8	90–100	FG	F	C	+++
19	MS + TDZ 1.0 + NAA 4.0	8	8	90–100	FG	F	C	+++
20	MS + TDZ 1.0 + NAA 5.0	8	8	90–100	FG	F	C	+++
21	MS + TDZ 1.0 + BAP 1.0	-	-	-	-	-	-	-
22	MS + TDZ 1.0 + BAP 2.0	-	-	-	-	-	-	-
23	MS + TDZ 1.0 + BAP 3.0	-	-	-	-	-	-	-
24	MS + TDZ 1.0 + BAP 4.0	-	-	-	-	-	-	-
25	MS + TDZ 1.0 + BAP 5.0	-	-	-	-	-	-	-

Values are means ± SD from three replicates. Note: No callus (−), Scanty callus (+), Moderate callus (++), profuse callus (+++). F friable, DG dark green, FG fresh green, SG snowy green, SLG snowy light green.

**Table 2:** Quantification of the main phytochemicals accumulated in twelve *I. rugosus* callus sample extracts (culture conditions are presented in Table S1).

Sample	CA (µg/g DW)	RA (µg/g DW)	BA (µg/g DW)	OA (µg/g DW)	PA (µg/g DW)
Ir#1	614.8 ± 20.2	1074.7 ± 18.9	98.0 ± 14.3	536.2 ± 18.8	454.8 ± 39.1
Ir#2	488.4 ± 24.6	751.6 ± 24.1	91.8 ± 19.5	201.7 ± 5.3	113.7 ± 33.9
Ir#3	784.0 ± 14.8	1519.5 ± 17.8	104.3 ± 18.8	348.4 ± 16.3	304.6 ± 33.0
Ir#4	735.6 ± 26.9	1158.3 ± 27.1	141.8 ± 16.3	317.1 ± 19.5	207.6 ± 28.2
Ir#5	728.2 ± 7.6	1685.2 ± 44.7	132.5 ± 24.8	631.0 ± 24.8	379.7 ± 14.2
Ir#6	979.5 ± 12.1	1259.0 ± 27.5	66.7 ± 9.4	198.2 ± 9.4	116.8 ± 23.6
Ir#7	575.6 ± 20.7	1279.0 ± 9.0	54.2 ± 5.4	389.0 ± 19.6	132.5 ± 18.8
Ir#8	901.6 ± 10.3	1708.9 ± 57.1	85.7 ± 9.1	386.0 ± 24.8	207.6 ± 8.8
Ir#9	647.2 ± 19.8	936.7 ± 13.1	22.9 ± 5.8	204.4 ± 23.6	69.9 ± 14.3
Ir#10	779.3 ± 18.0	797.1 ± 37.0	91.8 ± 10.8	248.3 ± 14.3	145.0 ± 23.6
Ir#11	886.8 ± 24.2	2013.5 ± 18.7	171.2 ± 9.2	331.2 ± 16.4	313.8 ± 14.1
Ir#12	835.8 ± 9.9	1335.9 ± 67.2	145.4 ± 5.1	429.8 ± 23.9	429.8 ± 14.3

Values are means ± standard deviations (*n* = 3).

**Table 3:** Antioxidant activities of 12 *I. rugosus* callus sample extracts. (Culture conditions are presented in Table S1).

Sample	DPPH (TEAC)	ABTS (TEAC)	ORAC (TEAC)	FRAP (AEAC)	CUPRAC (AEAC)	Chelation (µmol Fe^2+^)
Ir#1	674.4 ± 4.7	585.5 ± 9.0	421.8 ± 23.1	285.4 ± 6.3	260.3 ± 4.7	31.6 ± 0.9
Ir#2	474.4 ± 13.5	434.5 ± 15.4	306.7 ± 18.5	211.9 ± 3.2	193.3 ± 8.4	23.0 ± 1.4
Ir#3	911.7 ± 14.2	798.8 ± 15.1	529.4 ± 18.3	393.4 ± 6.9	354.2 ± 8.7	41.6 ± 1.0
Ir#4	721.2 ± 5.9	659.6 ± 25.1	453.2 ± 22.3	318.1 ± 6.4	273.5 ± 7.1	33.8 ± 1.7
Ir#5	1005.5 ± 13.8	879.5 ± 60.1	624.8 ± 9.0	456.8 ± 2.6	401.3 ± 2.7	45.2 ± 2.7
Ir#6	779.4 ± 5.4	708.1 ± 9.3	470.9 ± 14.5	354.8 ± 13.6	312.5 ± 8.8	34.3 ± 2.5
Ir#7	780.8 ± 6.2	688.2 ± 8.2	466.8 ± 9.6	349.3 ± 12.3	277.8 ± 8.4	34.9 ± 3.3
Ir#8	1043.2 ± 15.9	945.8 ± 6.4	641.0 ± 11.5	475.3 ± 10.0	410.8 ± 7.8	47.3 ± 1.5
Ir#9	563.1 ± 15.3	522.4 ± 5.2	350.1 ± 5.5	251.3 ± 9.0	235.7 ± 10.7	26.6 ± 2.8
Ir#10	516.3 ± 9.2	444.7 ± 18.2	318.1 ± 13.0	231.0 ± 8.8	186.1 ± 13.4	27.0 ± 2.8
Ir#11	1203.7 ± 53.2	944.7 ± 37.1	733.53 ± 7.3	535.8 ± 9.9	460.2 ± 5.5	54.8 ± 2.2
Ir#12	823.1 ± 25.6	727.1 ± 13.4	581.3 ± 173.5	353.8 ± 8.9	317.0 ± 4.8	35.9 ± 4.1

TEAC: Trolox C Equivalent Antioxidant Capacity (µM); AEAC: Ascorbic acid Equivalent Antioxidant Capacity (µM); Values are means ± standard deviations (*n* = 3).

**Table 4:** Anti-aging activities of 12 *I. rugosus* callus sample extracts expressed as percentage activities of control (DMSO) (culture conditions of the callus are presented in Table S1).

Sample	Elastase	Collagenase	Hyaluronidase	Tyrosinase	AGEs	SIRT1
Ir#1	77.8 ± 2.9	64.3 ± 3.0	85.8 ± 1.7	62.9 ± 2.3	78.5 ± 0.7	140.8 ± 5.0
Ir#2	90.7 ± 0.8	86.2 ± 2.2	88.4 ± 1.4	85.4 ± 1.7	79.8 ± 1.4	88.9 ± 3.3
Ir#3	79.8 ± 1.2	77.7 ± 3.0	88.6 ± 0.9	27.8 ± 12.1	84.4 ± 0.8	162.0 ± 7.0
Ir#4	83.0 ± 2.2	75.0 ± 3.3	87.7 ± 2.0	75.6 ± 2.6	77.6 ± 2.7	134.3 ± 10.3
Ir#5	76.8 ± 4.6	63.5 ± 4.3	80.1 ± 1.4	52.1 ± 4.4	73.8 ± 1.9	194.1 ± 6.4
Ir#6	87.9 ± 2.2	84.8 ± 0.3	85.6 ± 1.5	87.6 ± 1.9	76.1 ± 2.2	124.3 ± 11.0
Ir#7	85.8 ± 1.7	77.1 ± 1.9	86.6 ± 2.8	79.3 ± 1.4	76.9 ± 2.6	137.8 ± 5.8
Ir#8	85.9 ± 2.7	78.5 ± 0.9	89.2 ± 0.9	82.8 ± 1.7	83.2 ± 1.9	185.4 ± 11.0
Ir#9	90.2 ± 1.2	85.8 ± 1.6	88.7 ± 1.2	87.2 ± 1.5	83.7 ± 1.7	94.8 ± 4.2
Ir#10	86.2 ± 1.3	79.7 ± 1.3	91.5 ± 0.9	85.2 ± 1.1	85.1 ± 1.3	92.6 ± 3.2
Ir#11	79.2 ± 1.9	68.8 ± 2.1	78.7 ± 0.9	74.5 ± 2.7	70.8 ± 1.8	203.3 ± 6.2
Ir#12	74.7 ± 1.1	65.8 ± 2.2	77.8 ± 0.9	63.9 ± 2.5	65.9 ± 2.7	154.4 ± 7.9

Values are means ± standard deviations (*n* = 3).

**Table 5:** Pearson coefficient correlation linking the mains phytochemicals accumulated in *I. rugosus* callus extracts to their antioxidant and anti-aging activities.

	CA	RA	BA	OA	PA
DPPH	0.546	0.997 ***	0.471	0.477	0.537
ABTS	0.575	0.982 ***	0.484	0.447	0.466
ORAC	0.562	0.975 ***	0.511	0.550	0.604 *
FRAP	0.555	0.997 ***	0.447	0.423	0.510
CUPRAC	0.566	0.992 ***	0.454	0.466	0.513
Chelation	0.534	0.992 ***	0.456	0.465	0.559
Elastase	0.126	0.525	0.827 **	0.902 ***	0.748 *
Collagenase	0.097	0.571	0.900 ***	0.936 ***	0.720 **
Hyaluronidase	0.467	0.897 ***	0.572	0.602 *	0.538
Tyrosinase	-0.221	0.072	0.440	0.603 *	0.622 *
AGEs	0.608 *	0.943 ***	0.527	0.522	0.447
SIRT1	0.435	0.970 ***	0.665 *	0.646 *	0.625 *

* *p* < 0.05, ** *p* < 0.01, *** *p* < 0.001.

## Supplementary Materials

Supplementary materials can be found at http://www.mdpi.com/1422-0067/20/2/452/s1.

## Abbreviations

NAA	α-Naphthalene acetic acid
BAP	6-Benzyl adenine
TDZ	Thidiazuron
DPPH	2,2-Diphenyl-1-picrylhydrazyl
HPLC	High-performance liquid chromatography
CE	Callus Extract
WPE	Whole plant extract
MS	Murashige and Skoog
TPC	Total Phenolic Content
TFC	Total Flavonoid Content
DW	Dry Weight
FW	Fresh Weight
PGRs	Plant Growth Regulators
ROS	Reactive Oxygen Species
PAL	Phenylalanine ammonia-lyase
FRAP	Ferric reducing antioxidant power
CUPRAC	Cupric ion reducing antioxidant capacity
ORAC	Oxygen radical absorbance capacity
ABTS	2,2-Azinobis (3-ethylbenzthiazoline-6-sulphonic acid)
AGE	Advanced glycation end products
