# Supplementary material for: Isodon rugosus (Wall. ex Benth.) Codd In Vitro Cultures: Establishment, Phytochemical Characterization and In Vitro Antioxidant and Anti-Aging Activities
Source: Int J Mol Sci. 2019 Jan 21;20(2):452. doi: 10.3390/ijms20020452 (PMC6358864; doi:10.3390/ijms20020452)
Supplement: Supplementary file 1 [file ijms-20-00452-s001.pdf]

International Journal of *Molecular Sciences*

MDPI

Article

Efficient production of industrially attractive metabolites from *in vitro* cultures of *Isodon rugosus* (Wall. ex Benth.) Codd

Bilal Haider Abbasi[1,2,3,6,*,ψ], Aisha Siddiquah[1,ψ], Duangjai Tungmunnithum[2,3,4,ψ], Shankhamala Bose[6], Muhammad Younas[1], Laurine Garros[2,3,5], Samantha Drouet[2,3], Nathalie Giglioli-Guivarc'h[6], Christophe Hano[2,3,*,ψ]

[1] Department of Biotechnology, Quaid-i-Azam University, Islamabad-45320, Pakistan
[2] Laboratoire de Biologie des Ligneux et des Grandes Cultures (LBLGC EA1207), INRA USC1328, Plant Lignans Team, Université d'Orléans, 45067 Orléans CÉDEX 2, France
[3] Bioactifs et Cosmétiques, GDR 3711 COSM'ACTIFS, CNRS, 45067 Orléans CÉDEX 2, France
[4] Department of Pharmaceutical Botany, Faculty of Pharmacy, Mahidol University, 447 Sri-Ayuthaya Road, Rajathevi, Bangkok 10400, Thailand
[5] Institut de Chimie Organique et Analytique, ICOA UMR7311, Université d'Orléans-CNRS, 45067 Orléans CÉDEX 2, France
[6] EA2106 Biomolécules et Biotechnologies Végétales, Université de Tours, 37200 Tours, France

* Correspondence: BHA; bhabbasi@qau.edu.pk; CH; christophe.hano@univ-orleans.fr
ψ These authors contributed equally to this work.

Supplementary Materials:

Table S1: Culture conditions and explant origin of the 12 *I. rugosus* callus used for the determination of main phytochemicals, antioxidant and anti-aging activities

Table S2: Pearson coefficient correlation linking the main phytochemicals accumulated in *I. rugosus* callus extracts

Figure S1: Comparison of DPPH antioxidant activity of extracts at different PGRs from stem-derived callus culture (**A**) or leaf-derived callus culture (**B**).

Table S1.

Culture conditions and explant origin of the 12 *I. rugosus* callus used for the determination of main phytochemicals, antioxidant and anti-aging activities.

Sample	PGRs (mg/l)	Initial explant
Ir#1	TDZ (1.0)	Stem
Ir#2	TDZ (1.0)	Leaf
Ir#3	TDZ (2.0)	Stem
Ir#4	TDZ (2.0)	Leaf
Ir#5	TDZ (3.0)	Stem
Ir#6	TDZ (3.0)	Leaf
Ir#7	TDZ (1.0) + NAA (1.0)	Stem
Ir#8	TDZ (1.0) + NAA (1.0)	Leaf
Ir#9	TDZ (1.0) + NAA (2.0)	Stem
Ir#10	TDZ (1.0) + NAA (2.0)	Leaf
Ir#11	TDZ (1.0) + NAA (3.0)	Stem
Ir#12	TDZ (1.0) + NAA (3.0)	Leaf

Table S2.

Pearson coefficient correlation linking the main phytochemicals accumulated in *I. rugosus* callus extracts.

	**CA**	**RA**	**BA**	**OA**	**PA**
CA					
RA	0.506				
BA	-0.064	0.491			
OA	0.137	0.467	0.830**		
PA	0.263	0.525	0.520	0.718**	

* $p < 0.05$, ** $p < 0.01$, *** $p < 0.001$

Figure S1

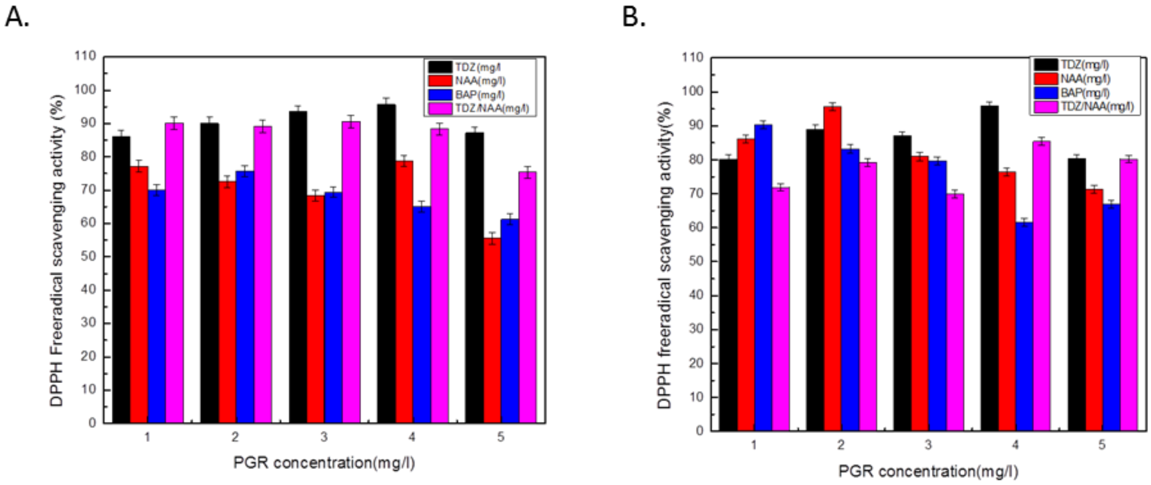

Figure S1: Comparison of DPPH antioxidant activity of extracts at different PGRs from stem-derived callus culture (**A**) or leaf-derived callus culture (**B**).

© 2019 by the authors. Submitted for possible open access publication under the terms and conditions of the Creative Commons Attribution (CC BY) license (http://creativecommons.org/licenses/by/4.0/).
